# Picornavirus Subversion of the Autophagy Pathway

**DOI:** 10.3390/v3091549

**Published:** 2011-08-26

**Authors:** Kathryn A. Klein, William T. Jackson

**Affiliations:** Department of Microbiology and Molecular Genetics, Medical College of Wisconsin, 8701 Watertown Plank Road, Milwaukee, WI 53226, USA; E-Mail: kklein@mcw.edu

**Keywords:** picornavirus, autophagy, membranes, viral replication

## Abstract

While autophagy has been shown to act as an anti-viral defense, the *Picornaviridae* avoid and, in many cases, subvert this pathway to promote their own replication. Evidence indicates that some picornaviruses hijack autophagy in order to induce autophagosome-like membrane structures for genomic RNA replication. Expression of picornavirus proteins can specifically induce the machinery of autophagy, although the mechanisms by which the viruses employ autophagy appear to differ. Many picornaviruses up-regulate autophagy in order to promote viral replication while some members of the family also inhibit degradation by autolysosomes. Here we explore the unusual relationship of this medically important family of viruses with a degradative mechanism of innate immunity.

## 1. Introduction

Autophagy is a cellular process by which cytosolic material is targeted for degradation by the cell [1]. Autophagy initiates when proteins, membranes, and organelles are engulfed into a unique double-membraned structure known as an autophagosome. This vesicle matures by acidification and fuses with lysosomes to form the degradative autolysosome [2]. Autophagy plays important roles in breaking down protein aggregates and cellular structures as well as recycling of intracellular components [3]. In addition to maintaining cellular homeostasis, autophagy can be induced by cellular stressors such as starvation, drug treatments, or infection. Autophagy is also as a mechanism by which the cell controls infection through degradation of cytosolic viruses and bacteria [4,5]. However, as is true for most immune responses, several pathogens subvert the autophagic machinery in order to promote their survival and replication.

In retrospect, the history of picornaviruses and autophagosomes stretches back to some of the earliest fine-detailed microscopic analyses of infected cells. For years, scientists have been learning how this medically important class of viruses rearranges cellular ultrastructure. In 1958, the first electron microscopy (EM) analysis was carried out by Marguerite Vogt’s group, using monkey kidney cells infected with poliovirus (PV) [6]. This study, one of the first EM studies conducted on a uniformly infected, synchronous population of infected cells, identified cytoplasmic vesicles termed by the authors “U bodies”. The presence of U bodies correlated with the maximal release of virus from the cells. As lysis approached, clear vacuoles were seen in place of the U bodies. The techniques used were insufficient to identify virions during infection. However, this work clearly established the unusual pattern of membrane rearrangements in the cytoplasm of picornavirus-infected cells.

In 1962, Samuel Dales and Richard Franklin published a fine structure study of murine L cells infected with encephalomyocarditis virus (EMCV) [7]. The images demonstrate cytoplasmic regions filled with multi-lamellar structures, which we would now identify as autophagosomes. This study marks the first demonstration that we can find of autophagic induction in a picornavirus-infected cell. Although large crystalline arrays of virus are observed in the cytoplasm, it is difficult to tell if any are within the membrane-bound cytoplasmic bodies.

In 1965, Dales, working with George Palade, published a comprehensive EM analysis of PV-infected cells [8]. The authors analyzed HeLa cells treated with PV at a high multiplicity of infection (MOI = 22) and used EM protocols that provided high-contrast images of intracellular membranes. In their images, Dales and Palade identified “membrane-enclosed cell bodies” occupying nearly the entire cytoplasm of the cell. They identified these bodies, which they measured to be 70–200 nm each in diameter, to be the same as the U bodies observed by Vogt. In the Dales study, progeny virus particles were observed both inside and outside the small bodies. These vesicles clearly have two lipid bilayers with an electron-light luminal region in between, a hallmark of classical autophagosomes. The authors suggested that newly formed viral particles were being taken up into these vesicles as a response to infection.

The question remained whether such vesicles are present or relevant during infections *in vivo.* It was not until 1984 that the neurons of PV-infected monkeys were examined at the level of electron microscopy [9]. The double-membraned vesicles seen in this *in vivo* study bear a strong resemblance to the vesicles identified by Dales and Palade in HeLa cells, and are strikingly similar to autophagosomes. This same study found double-membraned vesicles in PV-infected cultured cynomolgus monkey kidney (CMK) cells, indicating that the membrane rearrangements in cultured cells reflect *in vivo* intracellular rearrangements. Therefore, these membranes have been the subject of intense study for years.

Although the molecular tools to conclusively identify these membranes as autophagosomes would not be available for years, the evidence for double-membraned vesicle formation during picornavirus infection has been accumulating for more than half a century. Many questions remained—first and foremost being what role these vesicles play in the virus life cycle and the interaction between the virus and its host.

## 2. Membranes and Picornavirus RNA Replication

The predominant hypothesis for the role of autophagosomes in the viral life cycle is that they serve as a physical substrate for viral genomic RNA replication. All positive-sense RNA viruses replicate their RNA in association with cellular membranes, as has been extensively reviewed elsewhere [10]. The reason for this membrane association is unclear. The structure of the membrane-associated replication complexes varies from virus to virus, and it does not appear that the cellular origin of the membrane is important for RNA replication [11]. In fact, in at least one instance, retargeting the replication complex to a different cellular membrane seemed to promote increased RNA replication [12].

The majority of studies involving positive-strand RNA virus membrane rearrangements have focused on their putative association with RNA replication complexes. The nature of the membranes associated with viral RNA replication complexes differs among virus families and even among viruses within that family. Several flaviviruses, such as hepatitis C virus (HCV), use a “membranous web”, or “convoluted membrane”, as the site of RNA replication [13]. Severe acute respiratory syndrome (SARS) coronavirus replicates on a reticulovesicular network of membranes, including endoplasmic reticulum (ER)-derived vesicles [14,15]. For Semliki Forest virus, an alphavirus, replication complexes are found on modified lysosomes [16]. The nodavirus flock house virus replicates its genome on invaginations in the mitochondrial membrane [17].

For picornaviruses, the origin of the replication-associated membrane is not yet fully understood. There have been, to date, three hypotheses proposed for the membrane origin of picornavirus replication-associated membranes. One hypothesis, from studies of PV, suggests that vesicles resembling COPII secretory vesicles, which can be found in the cytoplasm following infection by PV, are the sites of RNA replication. These vesicles are marked with the COPII proteins Sec13 and Sec31 as well as the Arf1 GTPase complex, which regulates secretory transport [18,19]. In published images, these appear to be distinct from the double-membraned vesicles first seen by Dales and replicated in many subsequent studies, so the COPII-like vesicles may represent a separate class of membrane induced during infection [8].

The second hypothesis is that RNA replication takes place on small vesicles containing *trans*-Golgi markers, as observed for human parechovirus, Theiler’s murine encephalomyelitis virus, and one strain of human rhinovirus (HRV), HRV-1A [20–23]. It is presumed that these small vesicles derive from breakup of the Golgi apparatus during infection, and as yet there has been no follow-up work to elucidate the mechanism of their formation. There may be overlap between these vesicles and the COPII vesicles observed during PV infection.

**Figure 1.**
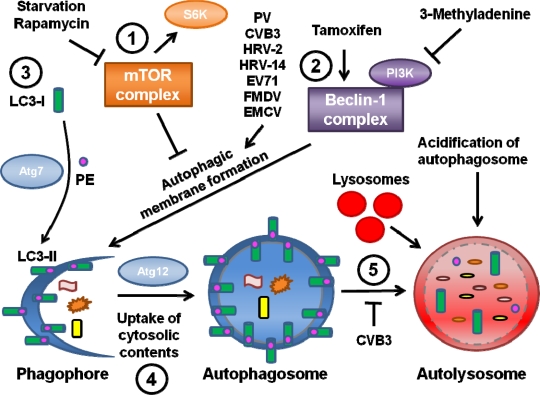
A simplified model of autophagy. **1.** The mTOR complex mTORC1 acts upstream of the autophagic pathway to suppress autophagic membrane formation, and mTOR activity can be monitored by detecting the activation of its downstream target S6 kinase. mTORC1 is inhibited when autophagy is induced via a cellular stress such as starvation or a drug treatment like rapamycin. **2.** After initiation of autophagy, a complex containing the Atg protein Beclin-1 is necessary for autophagosome formation. Tamoxifen induces autophagy by indirectly acting upon Beclin-1, while 3-MA suppresses autophagy due to its inhibition of PI3 kinases that interact with Beclin-1. The picornaviruses PV, CVB3, HRV-2, HRV-14, EV71, FMDV and EMCV have been shown to induce autophagic membrane formation. **3.** During autophagic membrane formation, the cytosolic Atg protein LC3-I becomes conjugated to PE, mediated in part by the ubiquitin E1-like enzyme Atg7, to form LC3-II. LC3-II proteins then specifically associate with a newly-formed crescent-shaped membrane termed a phagophore. The conversion of LC3-I to LC3-II has been observed with all of the picornaviruses discussed at length in this review. **4.** The phagophore elongates around cytosolic contents until the contents are completely sequestered within a fully formed double-membraned autophagosome. The formation of the autophagosome requires the ubiquitin-like protein Atg12. **5.** The autophagosome matures, becoming acidic, and fuses with lysosomes to form the degradative autolysosome. The cytosolic contents are degraded, and LC3-II is also degraded or recycled back to LC3-I. CVB3 appears to block this degradative step, while EMCV does not.

The third hypothesis is that viral RNA replication takes place on double-membraned vesicles derived from the autophagy pathway (Figure 1). These vesicles contain autophagy marker proteins as well as virus replication proteins. Since the autophagy pathway was found to promote PV replication, autophagosomes have been identified during infection by several other picornaviruses, including foot-and-mouth disease virus (FMDV), enterovirus 71 (EV71), EMCV, HRV, and coxsackieviruses. The data regarding autophagy during infection by each of these viruses is discussed in the next sections.

It is important to note that the three hypotheses for the origin of picornavirus RNA replication membranes are not mutually exclusive. HRV-1A, for example, does not induce autophagic signaling or autophagosomes [21]. This indicates that from virus to virus there is variation in the membrane-generating pathways induced, and that there is no universal picornavirus replication membrane. It is possible that autophagosomes contain Sec13, Sec31, and Arf1, unifying the two classes of vesicle seen during PV infection. It is also possible that there are two distinct classes of vesicle during infection, a COPII-derived vesicle and a vesicle derived from the autophagy machinery, each with distinct roles and identities. The remainder of this review focuses on the known connections between autophagy and picornavirus replication.

## 3. Poliovirus Subversion of the Autophagic Machinery

In order to understand the nature and origin of the observed vesicles, immuno-EM experiments were performed on PV-infected HeLa cells [24]. Initial experiments suggested that PV induced double-membraned autophagic-like structures 50–500 nm in diameter. While this is consistent with previous work, these structures are smaller than typical autophagosomes, which range from 500–1500 nm in diameter [8,25]. Using an antibody to the viral protein 2C to identify PV-relevant vesicles, cellular fractionation studies showed that these vesicles co-sediment with a variety of markers for ER, Golgi, and lysosomes [24,26]. Since autophagosomes contain markers from throughout the cell, these data led to the hypothesis that autophagy is involved in the formation of PV-induced membranes.

Confirmation of the nature of these vesicles had to wait for the identification of specific markers to monitor autophagosome formation, maturation and degradation. Genetic studies of the yeast *Saccharomyces cerevisiae* identified several genes, now termed ATG genes, essential for the autophagic pathway. Many of these genes are conserved in mammalian systems [27]. Although the signals leading to autophagic induction are still poorly understood, these studies have provided cellular protein markers for autophagosomal membranes and autophagic degradation, as shown in Figure 1.

Microtubule-associated protein light chain 3 (LC3), the mammalian homolog of yeast ATG8p, is a specific marker of autophagic membranes. LC3 is found in the cytoplasm when autophagy levels are low; this form is known as LC3-I. However, upon induction of autophagy LC3-I is conjugated to phosphatidylethanolamine (PE) and thereafter becomes membrane-bound to autophagosomes; this form is known as LC3-II (Figure 1, part 3) [28]. LC3-II appears to be required to complete formation of the autophagosome [29]. The isoforms of LC3 can be distinguished by Western blotting and a relative increase in LC3-II levels is indicative of increased autophagy. In addition, LC3 conjugated to GFP can be expressed in cells and monitored by immunofluorescence; LC3-GFP will form puncta in response to induction of autophagy. The ubiquitin-binding protein p62 also interacts with LC3-II in order to target cargo to autophagosomes for degradation [30]. Since p62 is degraded along with the rest of the autophagosome contents, steady-state levels of p62 can be monitored as an indicator of autophagic degradation levels. Advances in technology also allowed for the manipulation of the autophagic pathway via gene knockdown of various ATG proteins, the effects of which could be assessed by these newly available assays.

Using LC3 as a marker, studies were undertaken to investigate the PV-induced vesicles [31]. When GFP-LC3 was expressed in MCF-7 human breast cancer cells during PV infection, the GFP signal co-localized with the viral 3A protein, a component of viral replication complexes on host membranes [32]. Immunofluorescence showed that inducing autophagy using the pharmacological inducers rapamycin or tamoxifen when GFP-LC3 was expressed induced puncta indicative of autophagy.

During infection, punctate LC3 also co-localized with lysosomal-associated membrane protein LAMP1, a marker of late endosomes and lysosomes [31]. Studies of *Legionella pneumophila* had previously established co-localization of punctate LC3 with LAMP1 as an assay for identifying mature autophagosomes [33]. These data suggested that autophagosomes were forming and perhaps maturing in the presence of PV. When the viral proteins 2BC or 3A were individually expressed in human embryonic kidney (HEK) 293T cells, this co-localization was not observed. Only when the two proteins were co-expressed did LAMP1 co-localize with GFP-LC3.

When rapamycin or tamoxifen were present during infection of H1-HeLa cells the intracellular production of PV increased [31]. However, the autophagy inhibitor drug 3-MA decreased viral yield. In addition, RNAi directed against LC3 or ATG12 reduced both intracellular and extracellular viral titers. This study provided the first evidence that PV subverts the autophagic pathway in order to generate membranes for establishing viral replication complexes. The effect of inhibiting autophagy was more pronounced on levels of virus found in the medium prior to cell lysis than on cell-associated virus. Therefore, these data provided the first indication that the autophagy pathway plays a role in extracellular release of cytosolic contents. The existence of a non-conventional secretory pathway regulated by autophagy has now been confirmed using several other assays [34–36].

## 4. The Role of Autophagy during Infection by Other Picornaviruses

PV was the first picornavirus shown to subvert the autophagic machinery to promote its own replication, and several other picornaviruses have since been described to have a similar relationship with the autophagy pathway. Coxsackievirus group B (CVB), the most common cause of viral myocarditis, has also been reported to perturb the autophagic pathway in order to replicate on the surface of autophagosomes [37]. Ultrastructural analysis revealed that double-membraned vesicles were induced in HeLa or HEK 293T cells during infection with CVB3. Correspondingly, levels of LC3-II were significantly increased during infection. The GFP-LC3 signal re-localized to puncta in the presence of CVB3 as well, indicating that CVB3 infection induces autophagosome formation. Both viral titer and expression of the capsid protein VP1 decreased in the presence of 3-MA. Conversely, rapamycin treatment enhanced viral titer, and VP1 levels increased under starvation conditions. In addition, both VP1 expression and viral titer were reduced when the autophagy proteins Beclin-1, ATG7, and Vps34 were knocked down. The phosphorylation of eIF2α has been shown to be required for induction of autophagy; accordingly, the levels of phosphorylated eIF2α increased during viral infection [37,38]. However, p62 levels were not affected by CVB3 infection, indicating that degradation of autophagic targets was blocked. Furthermore, inhibition of the lysosome by LAMP-2 knockdown resulted in an increase in both VP1 levels and viral production. Taken together, this work suggests that CVB3 induces autophagic signaling to promote viral replication, but prevents autolysosome formation and autophagic degradation.

This research was extended *in vivo* using GFP-LC3 transgenic mice [39]. The murine pancreas was investigated because CVB3 reaches high viral load in this organ. When mice were infected with CVB3, the levels of LC3-II continually increased in the pancreas during the time course examined. The levels of p62 also built up over time, indicating that degradation was inhibited. In addition, GFP-LC3 re-localized to puncta that did not co-localize with LAMP1, suggesting that autophagic flux was blocked. Further observation revealed that, compared to uninfected controls, infected cells contained large GFP-LC3 positive structures. In addition, smaller vesicles were observed, leading the authors to distinguish between autophagosomes and the large structures, which were termed megaphagosomes. Ultrastructural analysis on CVB3-infected cells showed an abundance of small (∼250 nm) autophagosome-like vesicles similar to those observed during PV infection as well as more typically sized autophagosomes. Some megaphagosomes (2–3 μm) were also detected and appeared to have double membranes. Immuno-EM confirmed that LC3 was localized to the megaphagosome membrane. This work is consistent with the *in vitro* research in that CVB3 infection appears to induce autophagy and block autolysosome formation. However, more studies will need to be performed to investigate the link between viral induction of autophagy and viral replication *in vivo*.

CVB4 infection, tested in primary rat neurons, has been reported to have a similar relationship to autophagy as CVB3 [40]. LC3 was modified during infection and both autophagosome formation and viral production were suppressed in the presence of 3-MA. Calpain, a calcium-dependent cysteine protease, was also investigated for its role in CBV4-induced autophagy. Cleavage of spectrin, a calpain substrate, increased during viral infection and was inhibited in the presence of 3-MA. Calpain inhibitors caused a decrease in VP1 expression and viral production. Autophagosome formation was also depressed when infected cells were treated with the calpain inhibitors. This work demonstrated a role for calpain in CVB4-induced autophagy.

HRV, a major cause of the common cold, also frequently plays a role in asthma development and exacerbation. The species is divided into major and minor serotype groups, depending on the receptor utilized for cellular entry. HRV-14, a major group virus, and HRV-2, a minor group virus, have been shown to induce autophagy [31]. As with PV, infection with either HRV-2 or HRV-14 caused a co-localization of LAMP1 with punctate GFP-LC3 in MCF-7 cells. Further, monodansylcadaverine, a fluorophore retained in autophagosomes under gentle cell fixation conditions, showed a punctate pattern after infection with HRV14 that appeared similar to that observed during tamoxifen treatment. Finally, HRV-14 induced the formation of autophagosome-like vesicles. These data suggest that the HRVs examined induce autophagy. The effect of autophagy on HRV-2 replication is somewhat controversial, as a subsequent paper proposed that induction of autophagy did not affect HRV-2 viral production [41]. However, recent work showed that HRV-2 induces and subverts the autophagic pathway in order to promote viral replication [42]. Formation of GFP-LC3 puncta was observed following infection of HEK 293T cells with HRV-2. Further, modification of LC3 was detected during infection using Western blotting. Finally, induction of autophagy with rapamycin resulted in an increase in HRV-2 titers similar to that seen with PV, and a corresponding decrease was detected when autophagy was inhibited using 3-MA. This study confirmed the initial report that HRV-2 induces the autophagic pathway during infection and extended these findings to show that HRV-2 responds to the autophagic pathway in a manner similar to that of PV.

EV71 is one of the causative agents of hand-foot-and-mouth disease and is associated with neurological diseases in young children. One report provides evidence that EV71 induces autophagy that is associated with viral replication [43]. Upon infection with EV71, muscle or neuronal cell lines transfected with GFP-LC3 showed puncta formation indicative of autophagy, and EM analysis detected autophagosome-like vesicles of 100–500 nm in diameter in the presence of EV71. LC3-II levels also increased via Western blotting. Viral titers decreased in the presence of 3-MA and increased in the presence of inducers of autophagy, indicating that autophagy enhanced viral production. Furthermore, phosphorylation of both mTOR, a major effector of autophagic signaling, and its downstream target S6 kinase decreased during viral infection. Finally, experiments showed that mice infected with EV71 developed double-membraned structures in their cervical spinal cord neurons. By immuno-EM analysis, LC3 and the viral protein VP1 co-localized with these structures. Taken together, these data suggest that EV71 induces autophagy both *in vitro* and *in vivo* to facilitate viral replication, likely in a manner similar to that seen with PV infection.

FMDV, a highly infectious and contagious agricultural pathogen, has also been shown to exploit autophagy for viral replication [44]. During FMDV infection, GFP-LC3 signal re-localized from throughout the cytoplasm to the perinuclear region and co-localized with viral nonstructural proteins. The viral capsid protein VP1 co-localized with both GFP-LC3 puncta as well as the autophagy protein ATG5, which also appears to be in a punctate distribution during infection. Further, only during rapamycin treatment or FMDV infection did punctate GFP-LC3 co-localize with LAMP1. As seen with other picornaviruses, induction of autophagy using rapamycin enhanced viral production, whereas 3-MA treatment caused a reduction in viral production. Silencing either LC3 or ATG5 via siRNA also caused both intracellular and extracellular viral yield to be suppressed. Lastly, EM showed that formation of both single-membraned and double-membraned structures approximately 150 nm in diameter increased during FMDV infection; these structures co-localized with LC3 as well as the viral protein 2B. These results suggest that FMDV, like the previously discussed picornaviruses, induces the formation of autophagosome-like vesicles in order to enhance virus production.

EMCV, an agent of acute viral myocarditis, infects a wide range of hosts from rodents to humans but is particularly fatal in pigs. Recently published work details the role of autophagy in EMCV infection [45]. Utilizing a variety of rodent cell lines, EM showed that the number of autophagosome-like vesicles significantly increased during infection as compared to uninfected cells. LC3-II levels also increased upon infection, paralleling the EM results. The authors used UV-treated virus to show that active virus was required in order to generate the increase in LC3-II. Expression of GFP-LC3 re-localized from diffuse cytosolic staining to distinct puncta in the presence of the virus, and these puncta co-localized with the viral capsid protein VP1. The authors also observed a decrease in steady-state levels of p62 during EMCV infection. Treatment of the cells with E64-d, an inhibitor of cysteine proteases found in lysosomes, caused a rise in the levels of p62 as well as LC3, suggesting that EMCV was promoting autophagic degradation.

Treatment with rapamycin yielded significant increases in both intracellular and extracellular EMCV titers, and 3-MA treatment led to a loss in LC3 modification as well as a loss in GFP-LC3 puncta formation [45]. However, these treatments did not affect viral uptake or cell viability. siRNAs against either LC3 or ATG7 resulted in a reduction in intracellular and extracellular viral yields as well as a decrease in the levels of VP1 protein detected via Western. This work indicates that autophagy promotes EMCV replication. However, the mechanism may differ between PV and coxsackieviruses, as the replication of these viruses suffer in the presence of brefeldin A.

## 5. The Roles of Specific Viral Proteins in Induction of Autophagic Signaling

Most of what is known about the virus proteins involved in induction and regulation of autophagy comes from studies of PV. The contiguous region of the viral genome spanning the proteins 2B, 2C, and 3A is involved in altering the physical structure of the cell during virus replication. Previous studies have shown that expression of the viral protein 3A leads to severe swelling of the ER due to inhibition of the ER-Golgi trafficking, while the 2C protein plays a role in host membrane rearrangements and viral replication [46–48]. The 2C protein did not co-fractionate with the ER marker p63. When 3A was expressed singly or co-expressed with 2C, the ER marker calnexin did co-fractionate with these viral proteins [26]. It is possible that some host proteins may be excluded from these membranes due to modification by the viral proteins, but more research needs to be performed to investigate this phenomenon.

Expression of the viral precursor protein 2BC yielded single-membraned electron-light vesicles, in contrast to those induced by infection with PV [26]. Co-expression of 2BC and 3A not only showed membranes of a similar density to those induced by PV, but those membranes appeared to be autophagosome-like as well. This work not only provided a new method for examining autophagosomes, but identified the specific viral proteins that play a role in inducing autophagosome-like vesicles.

In addition to generating single-membraned vesicles, 2BC is sufficient to induce lipidation of cytosolic LC3-I to generate membrane-associated LC3-II [26,48]. Interestingly, expression of either 2B or 2C alone was not sufficient to increase LC3-II levels, indicating that a function of the 2BC precursor is required for this effect [49]. 2BC was among the first proteins identified shown to be sufficient for induction of autophagic signaling. This makes it all the more curious that classical double-membraned autophagosomes are not observed when 2BC is expressed alone. These data indicated for the first time that LC3-II formation is not sufficient for autophagosome formation. The specific role of 3A in forming autophagosomes is not yet understood.

Little is known about the proteins of other picornaviruses and their roles in inducing or regulating autophagy. When expressed in the absence of other viral proteins, the EMCV and FMDV 3A proteins each co-localize with GFP-LC3 [43,44]. These results are similar to those seen with PV 3A [31]. It has been shown that co-expression of FMDV 2B and 2C is sufficient to recapitulate certain functions of 2BC, but this is not the case for co-expression of PV 2B and 2C [49,50]. Therefore, the role of 2BC and its cleavage products may differ between FMDV and PV. It is unclear if the 2BC and 3A proteins of HRV-14 and CVB3 exhibit analogous functions during infection. More research is needed to fully understand how the picornaviruses utilize these proteins, perhaps in different ways, in order to induce and regulate autophagy.

## 6. The Purpose of Autophagy in the Picornavirus Life Cycle

The relationship between picornaviruses and autophagy appears to be unique among virus families. No other virus family has been consistently shown to subvert the autophagy pathway. Although some individual family members appear indifferent to autophagy, it is clear that the ability to subvert autophagy is shared by a large number of picornaviruses [21,41]. This suggests a common mechanism used by most species of the picornavirus family.

The specific role of autophagy in picornavirus replication remains a mystery. The presumption has been that the pathway plays an important role in generating membrane substrates for viral RNA replication. This remains to be formally demonstrated. In the case of PV, it remains to be seen whether the autophagosome-like and COPII-like vesicles identified by different groups are the same or unique, and whether they have separate or overlapping roles in promoting PV replication [8,18]. Coxsackievirus has been shown to inhibit autophagic degradation in a manner reminiscent of *L. pneumophila* [33,37,39]. This makes sense, as one can imagine the advantage to blocking degradation of nascent virions. However, during EMCV infection autophagic degradation increases, yet increasing autophagic signals still promotes production of infectious virus [45]. It is possible that autophagy is performing fundamentally different roles in the life cycles of coxsackievirus and EMCV. Alternatively, EMCV may have evolved a different mechanism to avoid degradation of infectious virus. It remains to be seen whether promoting or inhibiting autophagic degradation will prove to be the rule among picornaviruses.

The primary remaining question is, why autophagy? More specifically, what does this pathway uniquely provide to the *Picornaviridae*? There are several possibilities. The double-membraned nature of the vesicles is unique and may, for some reason, provide a superior substrate for viral RNA replication. Autophagosome membranes may contain proteins or lipids that promote genome production. The trafficking and localization of the autophagosome may be advantageous, either for RNA replication or a post-RNA step in producing infectious virus and delivering it to the extracellular space. Alternatively, there may be an advantage to subverting autophagy that we do not yet understand. The specific nature of the relationship between picornaviruses and autophagy will likely remain a focus of research for years to come.
